# Pancreatic resection with perioperative drug repurposing of propranolol and etodolac – the phase II randomized controlled PROSPER trial

**DOI:** 10.1007/s00423-025-03735-3

**Published:** 2025-05-22

**Authors:** Felix J. Hüttner, Rosa Klotz, Nathalia A. Giese, Bo Kong, Azaz Ahmed, Daniela Merz, Alexandra Pöchmann, Ina Burghaus, Thilo Hackert, Oliver Strobel, André L. Mihaljevic, Christoph W. Michalski, Markus W. Büchler, Markus K. Diener

**Affiliations:** 1 Department of General, Visceral and Transplantation Surgery, Heidelberg University Hospital, Im Neuenheimer Feld 420, 69120 Heidelberg, Germany; 2 Present Address: Department of General, Visceral and Thoracic Surgery, Klinikum Nürnberg, Prof.-Ernst-Nathan-Str. 1, 90419 Nuremberg, Germany; 3 Study Center of the German Surgical Society (SDGC), Im Neuenheimer Feld 130.3, 69120 Heidelberg, Germany; 4 Department of Medical Oncology, National Center for Tumor Diseases, Heidelberg University Hospital, Heidelberg, Germany; 5 Department of Cancer Immunology and Cancer Immunotherapy, German Cancer Research Center, Heidelberg, Germany; 6 Helmholtz Institute for Translational Oncology (HI-TRON), Mainz, Germany; 7 Coordination Centre for Clinical Trials (KKS), Medical Faculty & Heidelberg University Hospital, Heidelberg, Germany; 8 Present Address: Department of General, Visceral and Thoracic Surgery, University Hospital Hamburg-Eppendorf, Hamburg, Germany; 9 Present Address: Division of Visceral Surgery, Department of General Surgery, Medical University of Vienna, Vienna, Austria; 10 Present Address: Department of General, Visceral and Transplantation Surgery, University Hospital Tübingen, Tübingen, Germany; 11 Present Address: Botton-Champalimaud Pancreatic Cancer, Champalimaud Foundation, Lisbon, Portugal

**Keywords:** Pancreatic cancer, Perioperative period, Inflammation, Betablockade, COX-2-inhibition

## Abstract

**Purpose:**

The perioperative period is characterized by psychological stress and inflammatory reactions that can contribute to disease recurrence or metastatic spread. These reactions are mediated particularly by catecholamines and prostaglandins. The PROSPER trial aimed to evaluate whether a perioperative drug repurposing with a non-selective betablocker (propranolol) and a COX-2 inhibitor (etodolac) is feasible and safe in the setting of pancreatic cancer surgery.

**Methods:**

Patients undergoing partial pancreatoduodenectomy for pancreatic cancer were randomized to perioperative treatment with propranolol and etodolac or placebo. Main safety endpoint was the rate of serious adverse events (SAE) and the main feasibility endpoint was adherence. Overall and disease-free survival (DFS) as well as recurrences were assessed as efficacy parameters and the trial was accompanied by a translational study.

**Results:**

The trial was prematurely closed due to slow recruitment. 26 patients were randomized, but 6 never started trial medication. Finally, 9 patients received the trial medication and 11 patients placebo. There were 6 SAE in the treatment vs. 14 in the placebo group. Adherence was lower in the treatment group, but without statistically significance. Median DFS was 16.36 months (95%-CI 1.18 – not reached) in verum vs. 11.25 (95%-CI 2.2 – 17.25) in placebo group. The rate of distant recurrences was 11.1% in verum vs. 54.5% in placebo group.

**Conclusion:**

There were no safety concerns, but the trial intervention was not feasible given slow recruitment and limited adherence. However, the translational study and preliminary efficacy data revealed some promising findings, warranting further investigation.

**Registration:** DRKS00014054.

## Background

Pancreatic cancer is a devastating disease and in contrast to many other cancer entities its incidence and mortality have constantly been rising in recent years [1, 2]. Despite improvements in multimodal therapy of pancreatic cancer, the overall prognosis is still dismal [3]. Radical oncologic surgery remains the cornerstone of curatively-intended treatment of pancreatic cancer, however surgery is only possible in a subset of patients [4]. But even among those patients undergoing surgery, the risk of local or distant recurrence is high. Although a median overall survival of 54 months can be reached with modern multimodal treatment concepts, the median disease-free survival is only 21.6 months caused by distant metastatic recurrence in most of the cases [5].

There is evidence from preclinical and clinical studies that the perioperative period plays an important role in surgical treatment of cancer, which is currently not harnessed for cancer-directed therapy. This period spanning a few weeks from diagnosis (or end of neoadjuvant therapy) until the start of adjuvant therapy is characterized by psychological and physical stress and inflammatory responses [6]. These reactions are mainly mediated by an excessive release of catecholamines and prostaglandins. The resulting physiological changes can create a protumorigenic or prometastatic environment, potentially facilitating cancer progression or recurrence [7].

A combined treatment with betablockers and cyclooxygenase inhibitors (COX-inhibitors) in terms of drug repurposing promises an easy and effective approach to mitigate these effects [8]. Beta-blockers, such as propranolol, inhibit the effects of catecholamines, and COX-inhibitors, like etodolac, reduce the inflammatory response mediated by prostaglandins. The aim of the current randomized, placebo-controlled phase II trial was to assess whether a perioperative combined treatment with propranolol and etodolac is safe and feasible in patients undergoing partial pancreatoduodenectomy for pancreatic cancer.

## Methods

### Trial design

PROSPER was a randomized, patient and observer blinded, placebo-controlled, phase II trial, which was performed at the Department of General, Visceral and Transplantation Surgery of the University Hospital Heidelberg.

All relevant trial documents including the protocol were approved by the independent ethics committee of the University of Heidelberg and the competent authority, i.e. the German Federal Institute for Drugs and Medical Devices. The trial protocol was published as open-access publication [9] and the trial was registered before inclusion of the first patient in the German Clinical Trials Register (DRKS00014054). The trial was conducted in accordance with the Declaration of Helsinki [10], ICH-GCP, the German Drug Law (AMG), the European regulations Directive 2001/20/EC and Commission Directive 2005/28/EC and applicable data protection regulations and laws.

The current report was prepared in accordance with the recommendations of the CONSORT statement [11].

### Participants

The main eligibility criteria for trial participants are listed in Table 1.

**Table 1:** Main eligibility criteria

Inclusion criteria	Exclusion criteria
- Resectable malignancy of the pancreatic head, eligible for elective partial pancreatoduodenectomy in curative intent	- Any contraindication for partial pancreatoduodenectomy
- ECOG performance status 0–2	- Metastatic disease (Stage IV)
- Age ≥ 18 years	- Palliative resections
- ASA score ≤ III	- Extended resections (i.e. arterial resections, planned multivisceral resections)
- Ability to understand consequences of trial participation	- Acute or ongoing episode of cholangitis
- Written informed consent	- Acute or ongoing episode of pancreatitis
- Female subjects must be postmenopausal, surgically sterile, abstinent, or, if sexually active, be practicing an effective method of birth control and have a negative serum ß-hCG pregnancy test	- Chronic neuropathy
	- Renal failure (GFR < 50 ml/min/1,73 m2)
	- Liver cirrhosis
	- Atrioventricular block
	- Pregnant or breastfeeding women
	- Mental or organic disorders, which could interfere with giving informed consent or receiving treatments
	- Any contraindication to propranolol and/or etodolac

### Randomization and blinding

Patients were randomized in a 1:1 ratio to either perioperative treatment with propranolol + etodolac or placebo by block randomization with variable block sizes by means of a random list. The list was generated by personnel not otherwise involved in the trial. The hospital’s pharmacy manufactured consecutively numbered packages of trial medication of either verum or placebo according to the random list, which were delivered to the trial center in small batches on demand. If a new trial participant was included, the package with the lowest remaining number was handed over to the patient by an investigator or designated representative. Patients and trial staff including treating physicians/surgeons, outcome assessors, data collectors and data analysts were blinded to group assignment.

### Procedures

In the experimental group, patients received propranolol 2 × 20 mg/day for 10 days preoperatively, 2 × 40 mg on the day of surgery and one week thereafter and 2 × 20 mg/day during the second postoperative week. In addition, they received etodolac 2 × 400 mg/day during the same period of 25 days. In the control group, patients received placebo tablets/capsules of the same appearance, size and weight as propranolol and etodolac twice per day for a period of 25 days perioperatively starting 10 days before surgery. Partial pancreatoduodenectomy was performed according to local standards at the Department of General, Visceral and Transplantation Surgery of the University Hospital Heidelberg within a perioperative fast-track concept [12].

### Follow up

Thirteen trial visits were planned per patient including a screening visit, visits on the day before surgery, the day of surgery, postoperative days 1, 3, 5, 7, 14 (or day of discharge) and 30, as well as follow-up visits 3, 6, 12 and 24 months after surgery. A detailed trial schedule can be found in the published protocol [9].

### Outcomes

Outcome parameters consisted of safety endpoints, feasibility endpoints, efficacy endpoints and biological endpoints. The main safety parameter was the rate of serious adverse events (SAE) and serious adverse reactions (SAR) within a period of 3 months postoperatively. Furthermore, 30- and 90-day mortality, pancreas-associated morbidity including postoperative pancreatic fistula [13], delayed gastric emptying [14] and postpancreatectomy hemorrhage [15] according to the respective ISGPS definitions, postoperative biliary leakage according to the ISGLS definition [16] and postoperative intra-abdominal fluid collection or abscess were assessed. The main feasibility parameter was adherence to study medication quantified by measuring the percentage of actually taken trial medication from planned trial medication. In order to evaluate the possibility of implementing the intervention into routine treatment, completion of adjuvant chemotherapy was assessed as an additional measure of feasibility. Overall and disease-free survival (OS and DFS) as well as rates of local and distant recurrence were gathered as efficacy endpoints. Biological endpoints consisted of full blood count, C-reactive protein (CRP), albumin, creatinine clearance, bilirubin, international normalized ratio (INR), aspartate and alanine aminotransferase (AST and ALT), cancer antigen 19–9 (CA 19–9) and carcinoembryonic antigen (CEA).

### Translational part of the trial

The trial was amended by a translational part aimed at gaining deeper insights into the biological effects of the intervention. Peripheral blood samples were collected at several points of time as outlined in the published protocol [9]. Furthermore, a portal venous blood sample was taken during surgery to assess circulating tumor cells and a tissue biopsy of the tumor region as well as normal pancreatic tissue was taken from the resected specimen. The translational investigations included phenotyping of circulating immune cells by multicolor flow cytometry (fluorescence-activated cell sorting/FACS) analysis targeting specific immune cell populations (leucocytes, granulocytes, peripheral blood mononuclear cells (PBMCs), monocytes, lymphocytes, natural killer cells (NK-cells), B-cells, T-cells, CD4^+^T-cells, CD8^+^T-cells and natural killer T-cells (NKT-cells) with antibodies against CD3, CD4, CD8, CD14, CD16, CD20, CD25, CD45, and CD56 antigens as established by Oras et al. [17]. Furthermore, serum samples were analyzed for interleukin-6 (IL-6) levels by commercial ELISA kit (Thermo Fisher Scientific Inc) as IL-6 is a key cytokine mediating systemic inflammation and cancer progression [18].

Immunohistochemistry was conducted on 4 µM-sectioned paraffin-embedded tumor tissues using standard protocol with following steps: I) antigen retrieval with a citrate buffer at pH = 6, II) consequent blocking with peroxide and Universal Powerblock solutions, III) exposure to the primary antibodies (CD8, CD45, CD68) overnight at 4 °C, IV) washing and incubation with secondary antibodies and peroxide-diaminobenzidine(DAB)-based visualisation reagents from DAKO EnVision + System and V) counterstaining with haematoxylin and mounting of the slides. Whole-slide images were acquired with NanoZoomer 2.0 HT scan system (Hamamatsu, Hamamatsu City, Japan) and scanned at 20 × magnification. Quantification of immune cells was performed using a software-based image analysis system (HALO v3.5, Indica Labs, UK). The cell density in immune cell conglomerates was estimated as previously described [19]. Quantification algorithms were used individually for each distinctive staining protocol according to the intensity of DAB staining.

### Statistical analysis

Due to the exploratory nature of the trial, no formal sample size calculation was performed. Instead, the sample size was suggested by a panel of clinical and methodological experts at the investigators’ institution. A total of 80 patients (40 per group) was considered to be acceptable to assess safety and feasibility and to create first efficacy data for planning of a subsequent confirmatory trial. Thus, it was planned to randomize a total of 100 patients within the trial considering an estimated drop-out rate of 20%.

Since this trial had to be stopped prematurely due to insufficient recruitment, all analyses within this trial were performed in an exploratory fashion on the safety set comprising all subjects who received trial treatment at least once. In the safety set, patients were grouped according to study treatment they received.

The primary feasibility endpoint, i.e. adherence, was defined as relative amount of actual drug intake compared to planned drug intake. More precisely, the relative amount is the number of tablets/capsules taken divided by the planned number of tablets/capsules to be taken. If the planned number is lower than the taken number, the number planned is set to the taken one. Patients prematurely dropping out are included with 0 tablets/capsules for days thereafter. Patients were considered until death or premature closure of the trial or regular end. Sometimes a higher dose was administered than prescribed. To assess underdosing, prescribed was capped at administered, ignoring overdosing. Adherence was analyzed once descriptively and once using binomial regression with random effects.

The rate of SAEs was used as the primary safety variable. It was analyzed using a Poisson regression model using the time under risk as an offset and treatment group as an explanatory variable. The number of SAE within the relevant time interval was used as dependent variable, i.e. time from treatment start till visit 10 or last contact. The offset was log (time interval in months). A Wald confidence interval was calculated. In addition, the number of SAEs and the number of patients with at least one SAE was analyzed.

Overall survival since randomization and disease-free survival since surgery were analyzed using Kaplan–Meier methods. The two patients who did not have a surgery were excluded from the analysis of disease-free survival.

## Results

**Fig. 1:**
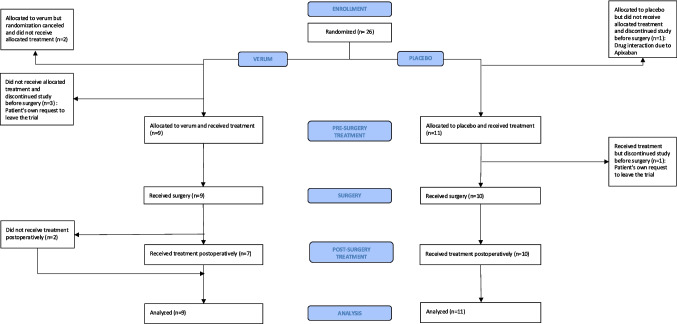
Trial flow chart (CONSORT)

This trial was prematurely closed due to feasibility reasons in terms of slow recruitment despite several adjustments of the trial design. A total of 1026 patients that presented to the outpatient clinic of the European Pancreatic Center at Heidelberg University Hospital between January 2019 and September 2020 were screened. Finally, 26 patients were randomized to perioperative treatment with propranolol + etodolac (*n* = 14) or placebo (*n* = 12). Six of them, five in the verum group and one in the placebo group, never started intake of trial medication. Thus, 20 patients received trial medication at least once and were included in the analysis. One patient in the placebo group terminated trial participation before surgery. The trial and patient flow is depicted in a CONSORT flow diagram (Fig. 1).

The baseline characteristics of the patients are provided in Table 2. Final histology showed pancreatic ductal adenocarcinoma (PDAC) in 15 patients. Other histological findings included one case of intestinal type papillary carcinoma (verum group), one case of neuroendocrine papillary carcinoma (placebo group), one case of main duct intraductal papillary mucinous neoplasia with high-grade dysplasia (placebo group) and one case of autoimmune pancreatitis type 1 (placebo group). A total of three patients could not be resected as planned, two patients (one in each group) due to local infiltration of the mesenteric root and one patient (verum group) was not resected, because previously undetected liver metastases were found during surgery. The median duration of surgery was 327 min (range: 91–610) and the median blood loss was 700 ml (range 50–1800). The median postoperative hospital stay was 14 days (range: 6–49). Pathologic results are displayed in Table 3.

**Table 2:** Baseline characteristics

N (%) or mean (SD)	Verum (*N* = 9)	Placebo (*N* = 11)	Total (*N* = 20)
**Gender**			
Male	4 (44·4%)	9 (81·8%)	13 (65·0%)
Female	5 (55·6%)	2 (18·2%)	7 (35·0%)
**Age (years)**	67·6 (7·4)	62·7 (14·4)	64·9 (11·8)
**Body mass index (kg/m**^**2**^**)**	23·8 (3·5)	24·6 (3·9)	24·2 (3·7)
**Neoadjuvant therapy**			
Yes	1 (11·1%)	0 (0·0%)	1 (5·0%)
No	8 (88·9%)	11 (100·0%)	19 (95·0%)
**Previous abdominal surgery**			
Yes	4 (44·4%)	5 (45·5%)	9 (45·0%)
No	5 (55·6%)	6 (54·5%)	11 (55·0%)
**Pre-existing exocrine insufficiency**			
Yes	3 (33·3%)	1 (9·1%)	4 (20·0%)
No	6 (66·7%)	10(90·9%)	16 (80·0%)
**Pre-existing diabetes mellitus**			
Yes	0 (0·0%)	4 (36·4%)	4 (20·0%)
No	9 (100·0%)	7 (63·6%)	16 (80·0%)
**Cardiac comorbidity**			
Yes	5 (55·6%)	2 (18·2%)	7 (35·0%)
No	4 (44·4%)	9 (81·8%)	13 (65·0%)
**Smoking status**			
Yes, current	1 (11·1%)	1 (9·1%)	2 (10·0%)
Yes, former	2 (22·2%)	6 (54·5%)	8 (40·0%)
No, never	6 (66·7%)	4 (36·4%)	10 (50·0%)
**Alcolohol abuse**			
Yes, current	1 (11·1%)	0 (0·0%)	1 (5·0%)
Yes, former	1 (11·1%)	2 (18·2%)	3 (15·0%)
No, never	7 (77·8%)	9 (81·8%)	16 (80·0%)

**Table 3:** Histopathologic results

N (%)	Verum (*N* = 9)	Placebo (*N* = 10)	Total (*N* = 19)
**Pancreatic ductal adenocarcinoma**			
ypT1b N1 R0	1 (11·1%)	0 (0·0%)	1 (5·3%)
pT2 N1 G2 R1	1 (11·1%)	1 (10·0%)	2 (10·5%)
pT2 N2 G2 R0	1 (11·1%)	1 (10·0%)	2 (10·5%)
pT2 N2 G3 R0	0 (0·0%)	1 (10·0%)	1 (5·3%)
pT2 N2 G3 R1	1 (11·1%)	0 (0·0%)	1 (5·3%)
pT3 N0 G2 R0	1 (11·1%)	0 (0·0%)	1 (5·3%)
pT3 N1 G3 R0	0 (0·0%)	1 (10·0%)	1 (5·3%)
pT3 N1 G3 R1	1 (11·1%)	0 (0·0%)	1 (5·3%)
pT3 N2 G2 R0	0 (0·0%)	1 (10·0%)	1 (5·3%)
pT3 N2 G3 R0	0 (0·0%)	1 (10·0%)	1 (5·3%)
Locally unresectable*	1 (11·1%)	1 (10·0%)	2 (10·5%)
Liver metastases	1 (11·1%)	0 (0·0%)	1 (5·3%)
**Other histopathology**			
Intestinal type papillary carcinoma pT2 N1 G2 R0	1 (11·1%)	0 (0·0%)	1 (5·3%)
Neuroendocrine papillary carcinoma pT2 N0 G3 R0	0 (0·0%)	1 (10·0%)	1 (5·3%)
Main duct IPMN with high-grade dysplasia	0 (0·0%)	1 (10·0%)	1 (5·3%)
Autoimmune pancreatitis type 1	0 (0·0%)	1 (10·0%)	1 (5·3%)

N = 19 in this table, because one patient in the placebo group terminated trial participation before surgery; *due to infiltration of the mesenteric root

The adherence to etodolac was lower in the treatment group with 78% (± 29%) adherence in the placebo group and 50% (± 26%) adherence in the verum group. This did not result in a statistically significant difference with an estimated odds ratio of 3.42 (95%-CI 0.93–12.49; *p* = 0.06). Similarly, the adherence to propranolol was 69% (± 37%) in the placebo group and 47% (± 28%) in the verum group, which did also not lead to a statistically significant difference with an estimated odds ratio of 2.42 (95%-CI 0.60–9.70; p = 0.20). The lower adherence to the trial medication occurred mainly in the postoperative period, whereas the intake during the 10 days preoperatively was similar with 94% (± 32%) in the placebo group vs. 89% (± 35%) in the verum group for etodolac and 87% (± 42%) vs. 91% (± 37%) for propranolol. Completion of planned adjuvant therapy was considered as another feasibility parameter. In the verum group, 5 of 8 patients (62.5%) regularly completed all cycles of adjuvant chemotherapy, one patient (12.5%) terminated adjuvant chemotherapy early and 2 patients (25.0%) did not receive adjuvant chemotherapy at all. In the placebo group, 3 of 11 patients (27.3%) completed all cycles of adjuvant chemotherapy, 3 patients (27.3%) terminated adjuvant chemotherapy early and 4 patients (36.4%) did not receive adjuvant chemotherapy at all and for the patient, who left the trial before surgery, the data were missing.

Regarding the safety parameters, there was no SAR and a total of 20 SAE, 14 in the placebo group and 6 in the verum group, within 3 months postoperatively. In the placebo group, 9 of the 11 patients (81.8%) suffered from at least one SAE compared to 5 of the 9 patients (55.6%) in the verum group. There was one fatal SAE, which occurred in the verum group in terms of a postoperative upper gastrointestinal bleeding on postoperative day 35. Since this patient stopped intake of trial medication upon the patient’s own request on postoperative day 10, a relation to study medication was judged as improbable. A detailed listing of all SAE is provided in the supplementary material. The SAE rate is 2.095 times as high in the placebo group as in the verum group (poisson regression, 95%-CI: 0.805–5.45, *p* = 0.1297). There were no substantial differences in postoperative morbidity and mortality between groups; the results are displayed in Table 4.

**Table 4:** Morbidity and mortality

N (%) or mean (SD)	Verum (*N* = 8)	Placebo (*N* = 10)	Total (*N* = 18)
**30-day mortality**	0 (0%)	0 (0%)	0 (0%)
**90-day mortality**	1 (12·5%)	0 (0%)	1 (5·6%)
**Delayed gastric emptying (ISGPS)**			
No	5 (62·5%)	8 (80·0%)	13 (72·2%)
Grade A	1 (12·5%)	1 (10·0%)	2 (11·1%)
Grade B	2 (25·0%)	1 (10·0%)	3 (16·7%)
Grade C	0 (0%)	0 (0%)	0
**Postoperative pancreatic fistula (ISGPS)**			
No	8 (100·0%)	6 (60·0%)	14 (77·8%)
G	0 (0%)	0 (0%)	0 (0%)
Grade B	0 (0%)	3 (30·0%)	3 (16·7%)
Grade C	0 (0%)	1 (10·0%)	1 (5·6%)
**Postpancreatectomy haemorrhage (ISGPS)**			
No	8 (100·0%)	9 (90·0%)	17 (94·4%)
Grade A	0 (0%)	0 (0%)	0 (0%)
Grade B	0 (0%)	0 (0%)	0 (0%)
Grade C	0 (0%)	1 (10·0%)	1 (5·6%)
**Biliary leakage (ISGLS)**			
No	8(100·0%)	10 (100·0%)	18 (100·0%)
**Intra-abdominal fluid collection/abscess**			
No	8(100·0%)	10 (100·0%)	18 (100·0%)

**Fig. 2:**
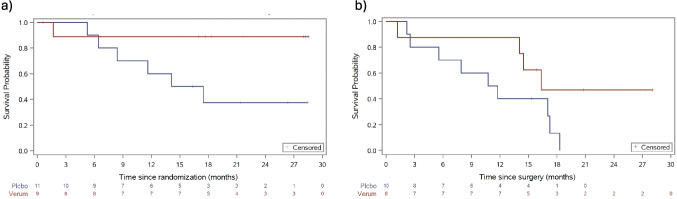
Kaplan–Meier curves for **a**) overall survival and **b**) disease-free survival. The analyzed safety set comprises 20 patients of whom 11 received Placebo and 9 received Verum

During the follow-up of the trial, only one patient died in the verum group and 6 in the placebo group. Median overall survival was not reached (95%-CI 1.68 months – not reached) in the verum group and 15.8 months (95%-CI 5.26 months – not reached) in the placebo group. Median disease-free survival was 16.36 months (95%-CI 1.18 – not reached) in the verum group compared to 11.25 (95%-CI 2.2–17.25) in the placebo group. Kaplan–Meier curves for OS and DFS are displayed in Fig. 2. The rate of local recurrence was 22.2% in the verum group compared to 18.2% in the placebo group. In contrast, the rate of distant recurrence was 11.1% in the verum group compared to 54.5% in the placebo group.

### Translational results

#### Therapy may help to improve short-term postoperative immunity

**Fig. 3:**
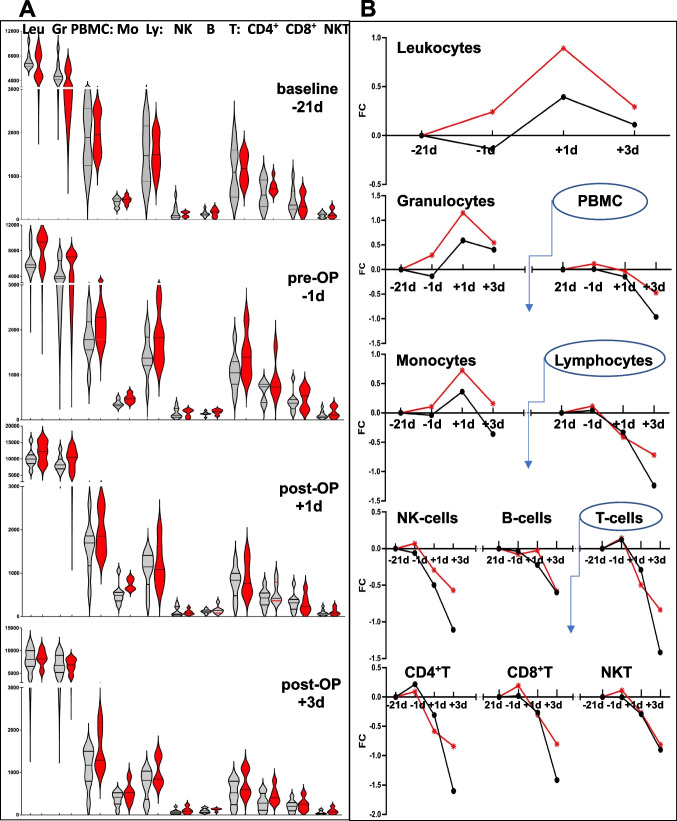
Multicolor flow cytometry (FACS) analysis; Placebo = grey; Verum = red. **A** Immunophenotyping of patients was performed by multicolor flow cytometry. The absolute numbers of circulating cells for each indicated (sub)-population has been calculated per µL blood and summarized per group in a form of a violin graph. **B** Time-resolved kinetics have been generated by normalizing measured counts to the individual baselines and consequent log2-transformation of the respective values. Data points are presenting mean values of placebo and verum groups

**Fig. 4:**
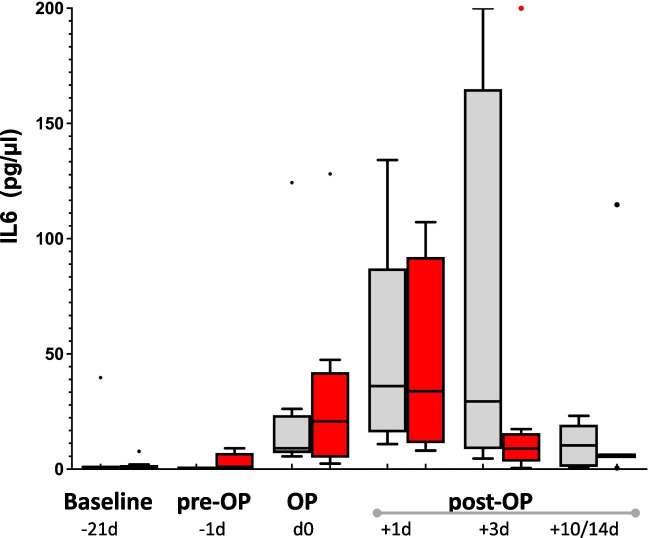
Box plot of IL-6 levels; Placebo (grey) and Verum (red) groups have been compared using Mann-Witney test

Multicolor flow cytometry (FACS) was performed to characterize immune cells in the blood of patients from placebo (*n* = 9) and verum (*n* = 6) group. According to these data, preoperative immunophenotype remained stable within 21 days in placebo group. Surgical intervention disturbed composition of circulating cells by causing transient myeloid boost (monocytes and granulocytes) while progressively deteriorating adaptive immunity (CD4 + and CD8 + T-lymphocytes, and B-lymphocytes), and also NK-cells within the next 3 days. The trial intervention did not change but ‘improved’ this pattern: it raised myeloid baseline during the preoperative phase and minimized postoperative loss of immune effectors (T- and NK-cells but not B- and NKT-cells) in the verum group (Fig. 3A-B). Remarkably, these positive reactions were associated with a lesser post-operative systemic inflammation. A measurement of the marker cytokine IL-6 in the serum of patients revealed a trend to more rapid post-operative normalization in the verum (*n* = 8) than in placebo (*n* = 8) group (Fig. 4).

In order to exclude pro-tumorigenic effects of the therapy, we also assessed portal venous blood for the presence of circulating tumor cells. However, no circulating tumor cells could be detected in both groups.

**Fig. 5:**
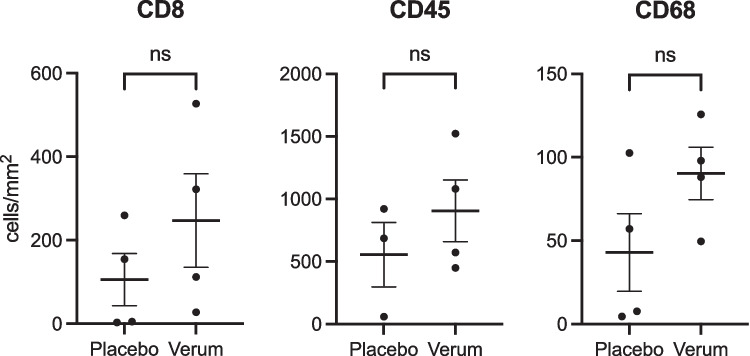
Immune cell quantification: Comparison of CD8 +, CD45 + and CD68 + cell densities between verum (*n* = 4) and placebo (*n* = 4) treated patients assessed by whole slide image analysis

To provide more insight into the potential treatment effect on the immunological composition of tumor microenvironment, relevant immune cells (CD8, CD45, CD68) were semi-automatically quantified on whole-slide images. Comparing the immune cell densities in the verum (*n* = 4) and placebo (*n* = 4) group did not show a statistically significant difference between the groups (Fig. 5). However, a consistent observation was the trend towards a higher density of all analyzed immune cells in the verum group, which indicates a modification of the local immune milieu towards (lymphocytic and monocytic) inflammation. The lack of statistical significance might be attributed to the small number of patients in this analysis.

## Discussion

PROSPER was the first phase-II trial to assess the effects of a perioperative drug repurposing of a non-selective betablocker (propranolol) and a COX-2 inhibitor (etodolac) in patients undergoing curative intent surgery for cancer of the pancreatic head. Unfortunately, the trial was unable to fulfil its recruitment goals and had to be prematurely closed, despite several adjustments of the trial design. Thus, the key finding of this pilot trial is that the intervention was not feasible within the current trial setting, although there were no safety concerns regarding the intervention. However, some interesting findings can be obtained by the results of the efficacy and translational analyses.

Looking at feasibility in more detail, there was insufficient recruitment on the one hand, but also a limited adherence to trial medication, particularly in the postoperative period, on the other hand. The main reasons for not recruiting potentially eligible patients were prior intake of betablockers, possible interactions with other medication or concerns by patients or the general practitioners regarding trial treatment. This limits the widespread application of the combination therapy with propranolol and etodolac substantially. In a retrospective analysis of the institutional pancreatic database at the University of Heidelberg, almost 30% of patients undergoing resection for pancreatic cancer received concurrent betablocker therapy [20] corroborating this frequent reason for exclusion. Regarding the reduced adherence in the postoperative period, it might have been cumbersome for patients to take 2 capsules (propranolol or placebo) + 2 tablets (etodolac or placebo) in the morning and in the evening in the early postoperative period after a partial pancreatoduodenectomy, since delayed gastric emptying is a frequent problem after partial pancreatoduodenectomy. This problem could be overcome by a single tablet preparation of verum and placebo in future trials, but currently there is no pharmaceutical company manufacturing this preparation to our knowledge. Furthermore, since the administration form was the same in the verum and placebo groups, this does not explain the relevant difference in adherence between both groups. Regarding the concerns that led to early termination of the trial medication intake or to not even starting it, an even closer communication with both, patients and their general practitioners, could have improved the feasibility and the compliance within this trial. Additionally, patients or patient representatives should most likely be involved already in the planning phase of a potential future trial to avoid such problems.

Regarding safety of the trial intervention, there were no concerns in terms of the main safety endpoint as there were no SAR observed. Moreover, the number of SAE was actually higher in the placebo group compared to the verum group. Furthermore, there were no relevant differences in postoperative mortality with only one death until postoperative day 90. Considering postoperative pancreas-specific complications, it is worth mentioning that there were no cases of postoperative pancreatic fistula in the verum group. Due to the small sample size, this might have been just caused by chance. However, this might also be attributed to an unexpected effect of the trial treatment – in particular the COX-2 inhibitor – on pancreatic secretion and warrants further investigation. The underlying mechanisms could be similar to the ones of NSAIDs in the prevention of post-ERCP pancreatitis, which is even recommended by current guidelines [21]. Other pancreas-specific complications did not show notable differences.

Considering the efficacy endpoints, interpretation needs to be very cautious, because of the limited sample size and the fact that not all patients had a pancreatic ductal adenocarcinoma on final histology and the tumor stages differed substantially. However, one could even argue that the pathology results were to the disadvantage of the verum group, since there were even two non-malignant cases in the placebo group and one patient with previously undetected liver metastases that could not be curatively resected in the verum group. Both, median overall and disease-free survival were longer in the verum group, but probably the most notable difference was the difference in distant recurrences with 54.5% in the placebo group and 11.1% in the verum group. This is supportive to the underlying mechanistic rationale of the trial intervention that the protumorigenic/prometastatic effect caused by an excess release of catecholamines and prostaglandins in the perioperative period can be effectively inhibited by propranolol and etodolac. In a similar trial by Haldar et al., in 34 patients with colorectal cancer there was also a markedly reduction of recurrences in the treatment group with propranolol and etodolac (12.5%) compared to the placebo group (33.3%) [22]. These findings further corroborate preclinical studies by the same group that the combined treatment with propranolol and etodolac may reduce metastases [23, 24]. Renz et al. also demonstrated the importance of catecholamines and beta-2-adrenergic signalling in PDAC in a preclinical study. Furthermore, they demonstrated in a cohort of 631 PDAC patients that the intake of a non-selective betablocker, such as propranolol, led to an improved overall survival compared to patients without betablocker therapy (40 vs. 23 months median overall survival) [25].

Regarding the translational part of the trial, not all investigations could be accomplished as planned, because of shortcomings in specimen sampling and quality. Furthermore, the results can only be interpreted very cautiously, due to the limited sample size. However, trial treatment seemed to cause a more rapid normalization of post-operative systemic inflammation in terms of faster normalization of IL-6 and mitigation of post-operative loss of immune effectors. Additionally, the quantification of immune cells in the tumor microenvironment was indicative of a modification of the local immune milieu towards increased peritumoral inflammation.

Despite of the strengths of the PROSPER trial in terms of its randomized, double-blind, placebo-controlled design, there are some limitations, which need to be considered in the interpretation of the findings. First, the sample size remained far behind the planned goals and thus, all analyses could only be conducted in an exploratory fashion. Second, adherence to the trial intervention was limited, which might have influenced its therapeutic effect. Third, the patient cohort was heterogeneous regarding the underlying diseases and disease stages on final pathology. Fourth, only the combined treatment with propranolol and etodolac was compared to placebo and we did not assess the effect of each drug as a monotherapy. However, this decision was based on previous research on this topic demonstrating the best effect for the combined treatment.

## Conclusion

In conclusion, although there were no safety concerns, a perioperative drug repurposing with propranolol and etodolac in the perioperative period surrounding partial pancreatoduodenectomy was not feasible within the current trial. Nonetheless, the exploratory results of the trial revealed some interesting findings, which merit further investigation in future projects. Apart from that, novel strategies for cancer-directed therapy in the perioperative period should be investigated in future trials.

## Data Availability

No datasets were generated or analysed during the current study.
